# Optimizing cardiovascular disease mortality prediction: a super learner approach in the tehran lipid and glucose study

**DOI:** 10.1186/s12911-024-02489-0

**Published:** 2024-04-16

**Authors:** Parvaneh Darabi, Safoora Gharibzadeh, Davood Khalili, Mehrdad Bagherpour-Kalo, Leila Janani

**Affiliations:** 1 Department of Biostatistics, School of Public Health, Iran University of Medical Sciences, Tehran, Iran; 2 Department of Epidemiology and Biostatistics, Pasteur Institute of Iran, Tehran, Iran; 3 Prevention of Metabolic Disorders Research Center, Research Institute for Endocrine Sciences, Shahid Beheshti University of Medical Sciences, Tehran, Iran; 4 Department of Epidemiology and Biostatistics, School of Public health, Tehran University of Medical Sciences, Tehran, Iran; 5 Imperial Clinical Trials Unit, School of Public Health, Imperial College London, London, UK

**Keywords:** Machine learning, Cox proportional hazard, Gradient boosting model, Support vector machine, Super learner, Tehran lipid and glucose study, Cardiovascular disease

## Abstract

**Background & aim:**

Cardiovascular disease (CVD) is the most important cause of death in the world and has a potential impact on health care costs, this study aimed to evaluate the performance of machine learning survival models and determine the optimum model for predicting CVD-related mortality.

**Method:**

In this study, the research population was all participants in Tehran Lipid and Glucose Study (TLGS) aged over 30 years. We used the Gradient Boosting model (GBM), Support Vector Machine (SVM), Super Learner (SL), and Cox proportional hazard (Cox-PH) models to predict the CVD-related mortality using 26 features. The dataset was randomly divided into training (80%) and testing (20%). To evaluate the performance of the methods, we used the Brier Score (BS), Prediction Error (PE), Concordance Index (C-index), and time-dependent Area Under the Curve (TD-AUC) criteria. Four different clinical models were also performed to improve the performance of the methods.

**Results:**

Out of 9258 participants with a mean age of (SD; range) 43.74 (15.51; 20–91), 56.60% were female. The CVD death proportion was 2.5% (228 participants). The death proportion was significantly higher in men (67.98% M, 32.02% F). Based on predefined selection criteria, the SL method has the best performance in predicting CVD-related mortality (TD-AUC > 93.50%). Among the machine learning (ML) methods, The SVM has the worst performance (TD-AUC = 90.13%). According to the relative effect, age, fasting blood sugar, systolic blood pressure, smoking, taking aspirin, diastolic blood pressure, Type 2 diabetes mellitus, hip circumference, body mss index (BMI), and triglyceride were identified as the most influential variables in predicting CVD-related mortality.

**Conclusion:**

According to the results of our study, compared to the Cox-PH model, Machine Learning models showed promising and sometimes better performance in predicting CVD-related mortality. This finding is based on the analysis of a large and diverse urban population from Tehran, Iran.

**Supplementary Information:**

The online version contains supplementary material available at 10.1186/s12911-024-02489-0.

## Introduction

Cardiovascular disease (CVD) is a class of disorders (such as heart failure, stroke, coronary heart disease, myocardial infarction) that affect the heart or blood vessels [1]. In the past few decades, CVDs, especially in undeveloped and developing countries, have become a major health threat [2, 3] by having the attribution of 32% of all global deaths, it has known as the leading cause of death worldwide [4]. A previous study shows that CVD-attributable deaths increased by at least 42% from 1990 to 2016 (12.3 million to 17.6 million attributed deaths, respectively) [5]. In terms of cost, CVD accounts for 7–21% of the direct and indirect costs of healthcare worldwide [6].

The rising prevalence of CVD underscores the urgency of identifying effective interventions to alleviate this global health burden. Over the decades, extensive efforts, exemplified by landmark studies such as the Framingham Heart Study, have been devoted to predicting CVD outcomes [7]. While traditional statistical regression models have formed the backbone of risk prediction [8], the landscape is evolving with the growing challenges of vast and complex datasets. In this context, the emergence of machine learning (ML) methods presents a promising avenue for refining CVD risk prediction [9–13]. ML, as a computer-based approach driven by algorithms, demonstrates notable efficacy in handling the intricacies of large datasets and has shown success in predicting various common diseases.

Despite the rapid growth of data and the surge in the number of features, there is a recognized need to develop prediction methods that can navigate these challenges. The application of ML methods has gained popularity due to their ability to discern complex patterns in data. Notably, several studies have illustrated the superior performance of selected ML methods in predicting outcomes across various medical domains, including postpartum hemorrhage [14], ischemic stroke readmission [15], and cardiovascular risk [16]. An influential study by Alaa et al. introduced an auto prognosis method, revealing that ML methods, such as Gradient Boosting Machine (GBM) and neural networks, outperform traditional Cox proportional hazard (Cox-PH) models in predicting cardiovascular disease risk [10].

In light of these advancements and the existing contradictions in the literature, our study aims to contribute to the understanding of the comparative performance of ML methods and Cox-PH models in prediction of CVD-related mortality. By addressing this gap in knowledge, we seek to provide insights that can inform more accurate risk assessments and guide decision-making in clinical practice.

In Iran, CVD has a huge impact on the health sector. Overall, 42% of annual deaths are related to CVD [17], and 1159.62$ million is spent annually on related costs [18]. Therefore, in the present study, we aimed to evaluate the performance of ML survival methods and determine the optimum model for predicting CVD-related mortality and identify the related risk factors in the presence of the censoring in an Iranian population.

## Methods

### Data

Tehran Lipid and Glucose Study (TLGS) [19–21] is a population-based cohort study designed by the research institute for endocrine sciences, Shaheed Beheshti University of Medical Sciences, to assess the risk factors of non-communicable diseases among an urban population under the coverage of three health centers in district number 13 of Tehran., Iran. This study consists of two major parts: 1- Determining the prevalence of cardiovascular disease and related risk factors (cross-sectional phase) and 2- Preventing the risk factors of the disease and improving lifestyle over the next 20 years (cohort phase). In total, between 1999 and 2001, 15,005 participants, aged ≥ 3 years, were recruited by a multistage cluster random sampling method from the TLGS. This dataset consists of a combination of interviews and laboratory tests for physical examinations. The interview data include demographic, socioeconomic, medical history, dietary, health-related, and physical function questions. All participants or their guardians signed a consent form before entering the study. For this study, individuals aged over 30 years were selected from phase 3 (2005–2008); these participants were followed through the subsequent three phases (phase 4, 2008–2011; phase 5, 2011–2014; phase 6, 2014–2017). For the purposes of this study, individuals were categorized as CVDs related death if they exhibited relevant conditions across any three consecutive phases. Alive participants who were lost to follow-up during the final phase were excluded from analysis due to uncertainty regarding their status by the end of the study period. Ultimately, among the 9258 subjects, 228 were identified as having died from CVDs.

### Study population

The research population is all people aged over 30 years in TLGS.

### Variable selection

According to WHO, National Health Service, and previous studies [22–24], twenty one major factors including age, sex, marital status, education, family history of stroke, smoking, physical activity, blood pressure (BP), total cholesterol (TC), type 2 diabetes mellitus (T2DM), body mass index (BMI), systolic blood pressure (SBP), diastolic blood pressure (DBP), weight, height,waist circumference, hip circumference, fasting blood sugar (FBS), triglyceride (TG), high-density lipoproteins (HDL) are considered as important risk factors for CVD. In addition to these variables, we also used some medications that have affected the CVD, such as Lipid lowering medications, beta-blockers, anti-hypertensive drugs, corticosteroids, and aspirin.

Of the 26 variables under investigation, 24 had missing values.To enhance ML algorithms performance, we employed six distinct methodologies for handeling missing data: complete case analysis, mean and mode imputation, amelia, mice, kNN, and missForest. Through a sensitivity analysis conducted, we observed that the missForest and MICE techniques notably improved algorithm performance. In alignment with Alsaber et al. [25] who demonstrated the efficacy of the random forest (RF) approach for handling missing values in machine learning (ML) methodologies, we adopted the missForest method for missing value imputation. This approach involves utilizing regression trees within a resampling framework to classify and impute missing data effectively [25]. Table 1 provides further information for each variable.

**Table 1:** Characteristic of variables included in the study

Variable	Description	# of missing (%)
Age	Years	0
Sex	Male/Female	0
Marital status	Married / Single / Divorced	4 (0.04)
Education	Primary / Secondary / Higher	766 (8.26)
Physical Activity	Low / Medium / High	416 (4.49)
Smoking	Current / Past / Never	237 (2.56)
Family History of Stroke	Dummy (Yes / No)	232 (2.50)
Diabetes Mellitus	Dummy (Yes / No)	60 (0.65)
Type 2 Diabetes Mellitus	Dummy (Yes / No)	829 (8.94)
Blood Pressure	Dummy (Yes / No)	255 (2.75)
Weight	Kg	631 (6.82)
Height	Meter (M)	560 (6.05)
Body Mass Index	Kg/M^2^	636 (6.86)
Hip circumference	Centimeter	637 (6.87)
Waist circumference	Centimeter	637 (6.87)
Systolic Blood Pressure	mm HG	224 (2.42)
Diastolic Blood Pressure	mmol/L	224 (2.42)
Total Cholesterol	mmol/L	277 (2.99)
Fasting Blood Sugar	mg/dl	283 (3.05)
Total Triglyceride	mmol/L	227 (2.99)
High-Density Lipoproteins	mmol/L	290 (3.13)
Lipid Lowering Drugs	Dummy (0 / 1)	0 (0.00)
Beta-Blockers	Dummy (0 / 1)	246 (2.65)
Antihypertensive Drug	Dummy (0 / 1)	0 (0.00)
Corticosteroid	Dummy (0 / 1)	239 (2.58)
Aspirin	Dummy (0 / 1)	241 (2.60)

### Variables and outcome definition

Hypertension was defined as the SBP ≥ 140 mmHg or DBP ≥ 90 mmHg [26], hypertriglyceridemia was determined as serum TGs ≥ 200 mg/dl and Low HDL-C as serum HDL < 40 mg/dl [27]. BMI was categorized as normal weight (18.5 ≤ BMI < 25 kg/m^2^), overweight (25 ≤ BMI < 30 kg/m2), and obese (BMI ≥ 30 kg/m^2^) [28].

Participants free of CVD at baseline were followed until the occurrence of a cardiovascular event, with the exact date of the event considered as the date of the end-point event. Alternatively, the follow-up continued until the participant’s death or until they were lost to follow-up, whichever came first. We considered the date of the last patient visit or the date of death due to a non-CVD event as censoring events.

### Variable selection strategy

To address overfitting resulting from a large number of covariates, particularly affecting the Cox-PH model [29], four distinct models were explored. In summary, the first model incorporates all features in their original scales, except for history of drug (reference model in statistical point of view). The second model focuses on CVD risk factors. The third model mirrors the second but substitutes waist-to-height ratio with waist-to-hip ratio. Lastly, the fourth, overemphasizes cardio-metabolic risk factors. For further details, please refer to Table 2.

**Table 2:** Model building strategies

Model	The feature considered
**Model 1**: All variables in their original scales besides the history of drugs	Age, Sex, Smoking status, Education, Marital Status, Family History of Stroke, SBP, DBP, BMI, Waist, Hip, FBS, TG, HDL, Physical Activity, Lipid Drug, Anti-Hypertension Drug, Aspirin, Corticosteroid.
**Model 2**: Transformed variables; the effect of changing the continuous to the discrete state of the features.	Age, Sex, Smoking status, Education, Marital Status, Family History of Stroke, Anti-Hypertension drug, BMI categories, Waist-to-Height Ratio, T2DM, high TG, low HDL, Physical Activity
**Model 3**: Transformed variables; the effect of changing the continuous to the discrete state of the features.	Age, Sex, Smoking status, Education, Marital Status, Family History of Stroke, Anti-Hypertension Drug, BMI categories, Waist-to-Hip Ratio, T2DM, high TG, low HDL, and Physical Activity
**Model 4**: Cardio-metabolic risk factors model; reducing the number of features.	Age, Sex, Smoking status, Education, Marital Status, Family History of Stroke, Cardio-metabolic risk factors*

*Cardio-metabolic risk factors refer to risk factors that increase the chance of experiencing cardiovascular events, such as age, sex, obesity, hypertension, dyslipidemia (high LDL cholesterol, high triglycerides, and low HDL cholesterol), dysglycemia, smoking, abdominal obesity, lack of consumption of fruits and vegetables, and sedentary lifestyle. Abbreviation: SBP: systolic blood pressure; DBP: diastolic blood pressure; BMI: body mass index; FBS: fasting blood sugar; TG: total triglyceride; HDL: high-density lipoprotein; T2DM: type 2 diabetes mellitus

### Data processing

Three steps were applied to make dataset ready for the analysis. Missing values were imputed using the missForest package [30]. In the next step, four different models are defined above, used to select the model that shows the best performance. The construction of all models involved the utilization of a development dataset through a 10-fold cross-validation approach, comprising 75 iterations. In each iteration, samples were selected randomly from the observed data using distinct seeds. Following the acquisition of the final models, an assessment, comparison, and reporting of their predictive performance were conducted using test datasets. To elaborate on the 10-fold cross-validation process and align with the recommendation by Dinh et al. [24], 80% of the development data were allocated for training purposes, while the remaining 20% were reserved for validation.

After pre-processing, Cox-PH and ML methods were fitted based on the training data and, then, the validity of the methods was examined based on testing data. In the end, according to the model selection criteria, the optimum model in predicting CVD-related mortality was identified.

### Models

- **Cox Proportional Hazard (Cox-PH) Model** is a semi-parametric model which assumes that independent variables have an exponential effect on the outcome and the log-hazard rate is a linear function of the covariates [31].
- **Machine learning (ML)** method in general is a computer-based approach that, by minimizing the error between observed and predicted outcomes, can learns all nonlinear and complex interactions between variables through pattern recognition and computational learning [16]. ML methods can be divided into supervised learning and unsupervised learning. Supervised learning, with focuses on classification, decomposes the dataset to identify differences between groups and learns a function to predict the outcome (it generally tries to estimate risk prediction), but unsupervised learning seeks to find a pattern or structure (such as clustering or grouping) in the data [32]. following ML methods were applied in this study:
- **Generalized Boosted Model (GBM)** is an ensemble prediction model, that based on the classification and regression relationships, trains weak learners to the best superior result by augment each other [33]. GBM optimizes the loss function using gradient descent and constructs the model based on the negative gradient of the previous loss function in an iterative cycle. The loss function is an important issue in GBM since the lower value of the loss function indicates a higher prediction performance [34].
- **Support Vector Machine (SVM)** with classification creates a decision boundary, hyperplane, between two classes. After creating the hyperplane, SVM tries to bring this boundary as close to the class points as possible. The greater the estimated distance between these boundaries, known as support vectors, indicates that the model predicts the event better [35].
- **Super Learner (SL)** is an ensemble algorithm which uses cross-validation to estimate the performance of multiple machine learning algorithms, or the same algorithm with different settings. It then creates an optimal weighted average of those algorithms using the test data performance. This approach has been proven to be asymptotically as accurate as the best possible prediction method that is tested [36, 37]. Among the advantages of SL are improved balance of covariates and reduced bias in case of serious model misspecification for treatment assignment [38].

Machine learning algorithms play a pivotal role in clinical decision-making, contributing to improved risk prediction, stratification, and treatment planning for CVD mortality. Clinicians can capitalize on the comprehensive approach of supervised learning for more accurate and robust predictions, facilitating enhanced risk stratification and treatment planning. Additionally, the ability of GBM to discern subtle patterns and nuanced risk factors contributes to the precision of risk assessments. Ultimately, SVM predictions aid in patient stratification, enabling the identification of individuals at higher risk and the customization of interventions based on their specific risk profiles [39].

### Dealing with multicollinearity, overfitting, and underfitting

A thorough examination of the features in the dataset was conducted to identify any high correlations. In instances where multicollinearity was observed, techniques such as variable scaling, dimensionality reduction, or, when necessary, removal of highly correlated features to mitigate its impact were applied. Additionally, regularization techniques, such as L1 or L2 regularization, were considered to penalize excessively large coefficients and improve model stability in the presence of multicollinearity. Several strategies to tackle overfitting and underfitting were applied. First, a 10-fold cross-validation approach was applied during the model development phase to assess the model’s performance on multiple subsets of the data. This helped identifying the optimal level of model complexity. Regularization techniques, such as dropout or weight decay, were also employed to prevent overfitting by penalizing overly complex models. Furthermore, we carefully tuned hyperparameters, utilizing techniques like grid search or random search, to find the optimal configuration that balanced model performance on the training and validation sets. Learning curves were also monitored to ensure that the model did not underfit the training data, and if necessary, the model’s complexity was adjusted to achieve a better fit.

#### Model selection and performance criteria

To assess the performance of survival models concordance index (C-index), Brier score (BS), prediction error (PE), and time-dependent area under the curve (TD-AUC) criteria were used [40–43].

##### C-index

is a rank-correlation measure between time point observations (in testing data) and predicted probability scores (in training data) [41]. This statistic, which is a generalized Tau-Kendall correlation method for censored data, has a range from 0 to 1. A value of 1 indicates a very good performance of the model in differentiating patients with different results (complete agreement) and a value of 0 indicates the inability of the model to separate patients (no agreement) [44]. In summary, C-index is a measure commonly used in survival analysis to evaluate the predictive accuracy of a model. It assesses the model’s ability to correctly order the predicted survival times of pairs of subjects. In the context of survival analysis, subjects are typically individuals or items that are followed over time to observe the time until a certain event occurs (e.g., time until failure or death).

##### Integrated brier score

is computed by integrating the Brier Score across distinct time intervals, offering a more accurate understanding of the model’s accuracy over the entire predefined timeline [45]. Brier Score is a quadratic score function that measures the accuracy of predictions, which can be calculated for survival outcomes using a weight function of the conditional probability of uncensored observations over time [40]. BS has a range from 0 to 1. With values greater than 0.25 indicate poor model performance, and lower values indicate better model prediction performance [46].

##### Prediction error

is a loss function that quantifies the absolute distance between predicted and observed time points of participants [42]. Lower value indicate the more reliable results [47].

##### Time-dependent area under the curve

is an efficient tool to evaluate the accuracy of diagnostic survival models. The range of TD-AUC is from 0 to 1 for each point time which the value of 1 reflects the perfect accuracy of the model in a specific time [48]. Overall, TD-AUC provides a nuanced view of how well a machine learning algorithm discriminates between positive and negative outcomes at different time points during its predictive horizon.

### Software and packages

In order to impute the missing data, the *missForest* package was used. *faraway* [49] and *tibble* [50] packages were used to separate the dataset into training and testing datasets. The *survival* [51] package was used to evaluate the specificity of follow-up time and fitting the Cox-PH model. GBM, SVM, and SL models were implemented by *gbm* [52], *survivalsvm* [53], and *survSuperLearner* [54] packages, respectively. Finally, to evaluate the performance of the models, the C-index, BS, PE, and TD-AUC were calculated using the *caret* [55] and *survAUC* [56] packages. All analyzes were conducted in R version 4.0.6 [57].

## Results

### Data

**Fig. 1:**
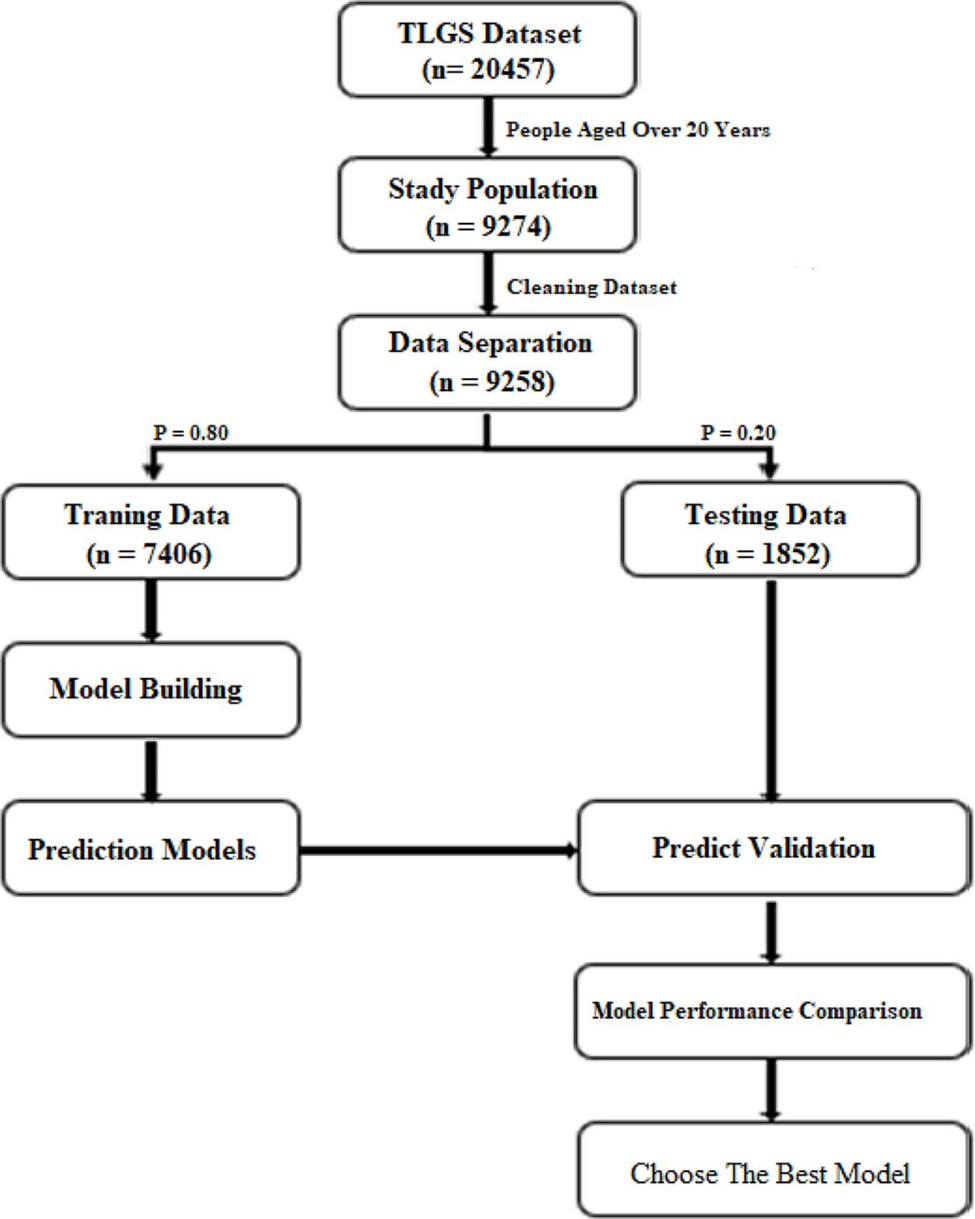
Processing and model selection. A flow diagram visualizing the TLGS dataset

Figure 1 shows the flow diagram from raw data through the best model selection. Of a total of 20,457 participants, 9274 participants met the eligibility criteria. After excluding 16 participants without any record of the outcome of interest, the analysis consisted of 9258 participants. To conduct the methods, the eligible dataset was randomly divided into training and validation subsets with a sample size of 7406 (80%) and 1852 (20%), respectively.

### Characteristics of participants

Out of 9258 participants with a mean age of (SD; range) 43.74 (15.51; 20–91), 56.6% were female. The proportion of CVD-related mortality was 2.5% (228 participants). Most of CVD-related mortality were married (83.77%), male (67.98%), older than 60 years (65.79%), and BMI greater than 30 (77.19%). The results of primary analysis showed that all variables except for physical activity, family history of stroke, weight, height, and HDL might not have a potential effect on CVD-related mortality (*P* > 0.05). More details are presented in Table 3.

**Table 3:** Characteristics of participants aged over 30 years in the TLJS study cohort

Variables	Total (*n* = 9258)	Alive (*n* = 9030)	Death (*n* = 228)
Categorical; Count (%)			
Age * > 40* * 40–60* * > 60*	4082 (44.09) 3561 (38.46) 1615 (17.44)	4075 (45.13) 3490 (38.65) 1465 (16.22)	7 (3.07) 71 (31.14) 150 (65.79)
Sex * Male* * Female*	4018 (43.40) 5240 (56.60)	3863 (42.78) 5167 (57.22)	155 (67.98) 73 (32.02)
Marital Status Single Married Divorced	1415 (15.29) 7181 (77.60) 658 (7.11)	1412 (15.64) 6990 (77.44) 624 (6.91)	3 (1.32) 191 (83.77) 34 (14.91)
Education Primary Secondary Higher	2087 (24.55) 5003 (58.85) 1411 (16.60)	1997 (23.96) 4943 (59.30) 1396 (16.75)	90 (54.55) 60 (36.36) 15 (9.09)
Physical Activity Low Medium High	3392 (38.36) 3940 (44.56) 1511 (17.09)	3306 (38.25) 3858 (44.64) 1479 (17.11)	86 (43.00) 52 (41.00) 32 (16.00)
Smoking Non-smoking Former Current	7025 (77.87) 856 (9.49) 1141 (12.65)	6894 (78.32) 819 (9.30) 1089 (12.37)	131 (59.55) 37 (16.82) 52 (23.64)
Family History of Stroke Yes No	932 (10.32) 8095 (89.67)	902 (10.24) 7905 (89.76)	30 (13.64) 190 (86.36)
Type 2 diabetes mellitus Yes No	1046 (12.40) 7387 (87.60)	949 (11.54) 7274 (88.46)	97 (46.19) 113 (53.81)
High Blood pressure Yes No	1745 (19.38) 7259 (80.62)	1616 (18.39) 7169 (81.61)	129 (58.90) 90 (41.10)
BMI Underweight Normal weight Overweight Obese	161 (1.74) 2614 (28.24) 3543 (38.76) 2940 (31.76)	161 (1.78) 2562 (28.37) 3451 (38.22) 2856 (31.63)	0 (0.0) 52 (22.81) 92 (40.35) 84 (36.84)
High Triglyceride Yes No	3906 (43.48) 5077 (56.52)	3780 (43.15) 4981 (56.85)	126 (56.76) 96 (43.24)
Low High-density lipoprotein Yes No	6993 (77.96) 1977 (22.04)	6823 (77.97) 1928 (22.03)	170 (77.63) 49 (22.37)
Lipid Drug Yes No	271 (2.93) 8987 (97.07)	246 (2.72) 8784 (97.28)	25 (10.96) 203 (89.04)
Beta-Blockers Yes No	862 (9.31) 8396 (90.69)	808 (8.95) 8222 (91.05)	54 (23.68) 174 (76.32)
Anti-Hypertension Drug Yes No	692 (7.47) 8566 (92.53)	625 (6.92) 8405 (93.08)	67 (29.39) 161 (70.61)
Corticosteroid Yes No	94 (1.02) 9164 (98.98)	90 (1.00) 8940 (99.00)	4 (1.75) 224 (98.25)
Aspirin Yes No	550 (5.94) 8708 (94.06)	484 (5.36) 8546 (94.64)	66 (28.95) 162 (71.05)
Continuous; Mean (SD)			
Age (year)	43.74 (15.51)	43.23 (15.25)	63.87 (11.77)
Weight (kg)	71.92 (13.56)	71.90 (13.58)	72.78 (12.75)
Height (cm)	162.42 (9.67)	162.45 (9.65)	161.20 (10.04)
Body mass index (kg/m^2^)	27.29 (4.85)	27.27 (4.86)	28.03 (4.48)
Hip circumference (cm)	101.65 (9.18)	101.68 (9.19)	100.24 (8.95)
Waist circumference (cm)	91.26 (12.58)	91.08 (12.57)	98.49 (10.46)
Systolic blood pressure (mmHg)	116.92 (18.92)	116.39 18.41)	137.99 (25.78)
Diastolic blood pressure (mmHg)	74.77 (10.71)	74.64 (10.60)	79.95 (13.52)
Cholestrrol (mg/dL)	192.02 (41.88)	191.62 (41.81)	207.86 (41.85)
Fasting blood suger (mg/dL)	98.33 (32.12)	97.45 (30.38)	132.78 (64.05)
Triglyceride (mg/dL)	160.18 (106.96)	159.59 (106.56)	183.44 (119.60)
High-density Lipoprotein (mg/dL)	39.19 (10.43)	39.22 (10.41)	38.08 (11.11)

### Variable selection and comparison of models’ prediction accuracy

Table 4 shows results of the selection process of four models based on study methods (Cox-PH, GBM, SVM, and SL). Accordingly, although all the models have provided favorable results, models I and III were used to check the efficiency of the methods. The reason for choosing model I was to be in alignment with other studies and the reason for choosing model III was the difference in the efficiency of SVM, so that the efficiency of this method has a drastic difference compared to model I ($$ {AUC}_{model I}$$ = 85.25 and $$ {AUC}_{model III}$$ = 90.13).

**Table 4:** Four clinical models: the strategy of selecting the best scale of variables

Method	Clinical Model	Integrated Brier Score	Prediction Error	C-index	AUC (CI)
Cox-PH	1	0.011	0.011	90.03	**93.83** (93.12–94.54)
	2	0.011	0.012	90.21	93.42 (92.71–94.13)
	3	0.011	0.011	**91.47**	93.48 (92.77–94.19)
	4	0.011	0.012	88.06	91.97 (91.26–92.68)
GBM	1	0.013	0.013	89.79	90.52 (89.81–91.23)
	2	0.013	0.013	90.14	91.14 (90.43–91.85)
	3	0.013	0.013	**90.18**	**91.67** (90.96–92.38)
	4	0.013	0.013	86.52	87.25 (86.54–87.96)
SVM	1	0.014	0.013	75.99	85.25 (84.54–85.96)
	2	0.014	0.013	89.10	90.01 (89.30–90.72)
	3	0.014	0.013	**89.63**	**90.13** (89.42–90.84)
	4	0.014	0.013	88.95	89.95 (89.24–90.66)
SL	1	0.011	0.011	91.60	**94.34** (93.63–95.05)
	2	0.011	0.011	90.55	93.59 (92.88–94.30)
	3	0.011	0.011	**92.81**	93.73 (93.02–94.44)
	4	0.012	0.011	87.97	91.86 (91.15–92.57)

Model I included Age, Sex, Smoking status, Education, Marital Status, Family History of Stroke, SBP, DBP, BMI, Waist, Hip, FBS, TG, HDL, Physical Activity, Lipid Drug, Anti-Hypertension Drug, Aspirin, Corticosteroid; Model III included Age, Sex, Smoking, Education, Marital Status, Family History of Stroke, Anti-Hypertension Drug, BMI categorization, Waist-to-Hip Ratio, T2DM, high TG, low HDL, and Physical Activity. GBM = Gradient boosting model; SVM = support vector model; SL = super learner; CI confidence interval

**Fig. 2:**
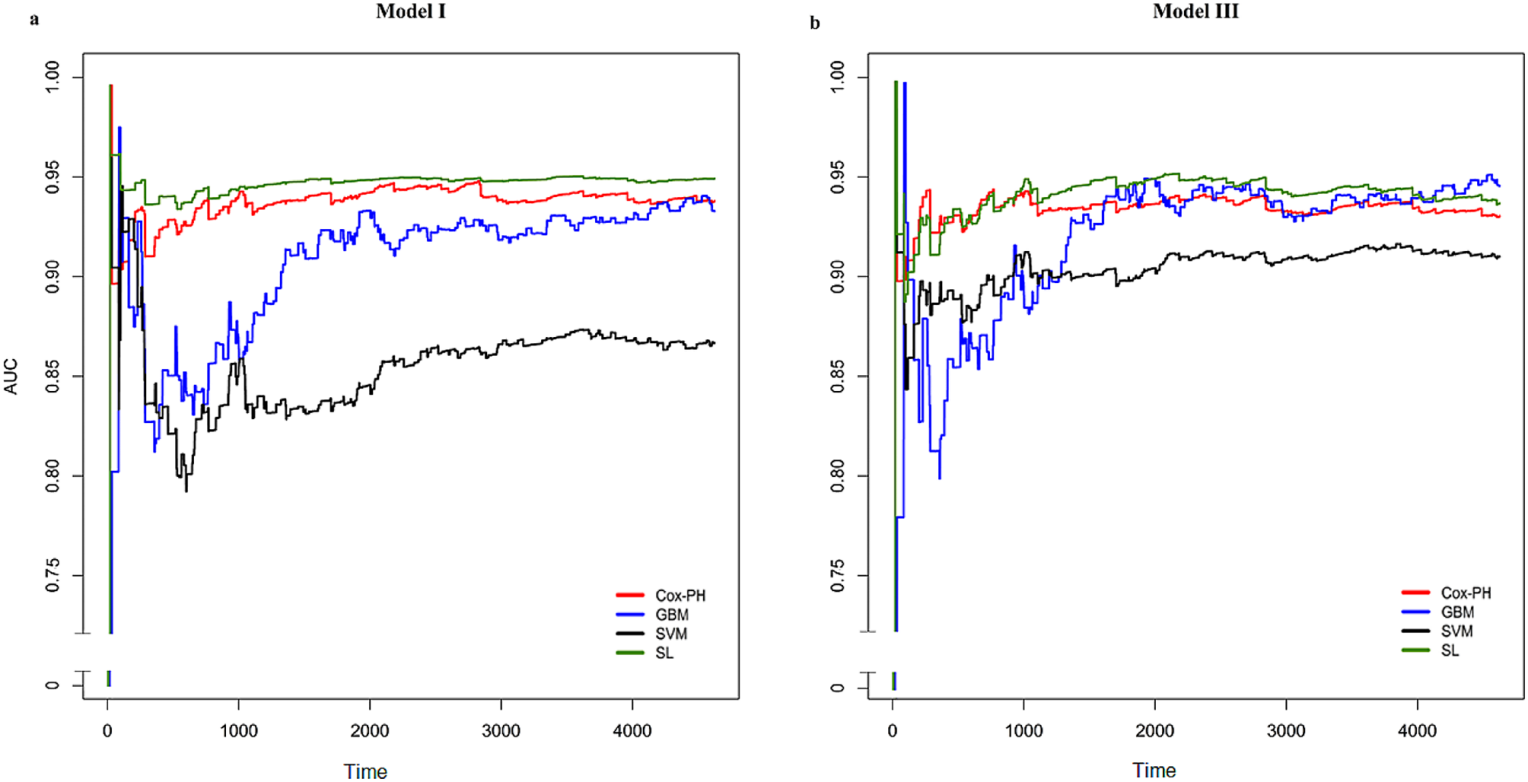
Comparison of models Prediction accuracy based on TD-AUC of model I (**a**) and model III (**b**) Model I included Age, Sex, Smoking, Education, Marital Status, Family History of Stroke, SBP, DBP, BMI, Waist, Hip, FBS, TG, HDL, Physical Activity, Lipid Drug, Anti-Hypertension Drug, Aspirin, Corticosteroid; Model III included Age, Sex, Smoking, Education, Marital Status, Family History of Stroke, Anti-Hypertension Drug, BMI categorization, Waist-to-Hip Ratio, T2DM, high TG, low HDL, and Physical Activity

In model I, the SL had maximum of TD-AUC (94.34%), followed by Cox-PH with 93.83% and GBM with 90.52%. The lowest TD-AUC also belonged to SVM (85.25%) (see Fig. 2A). This ranking remains valid for C-index (91.60%, 90.03%, 89.76%, and 75.99%, respectively). Regarding the BS and PE, SL and Cox-PH had almost the same performance (0.011: both criteria were the same up to three decimal points). On the other hand, the SVM showed BS and PE equal to 0.014 and 0.013, respectively, had the lowest prediction among other methods.

In model III, SL had the highest TD-AUC (94.34%) and C-index (92.81%), followed by Cox-PH (TD-AUC = 93.83%, C-index = 91.47) and GBM (TD-AUC = 91.67%, C-index = 90.18%) (see Fig. 2B). The lowest TD-AUC and C-index also belonged to SVM with 90.13% and 89.63%, respectively). Such as Model I, BS and PE are the same in SL and Cox-PH, and SVM has the highest BS and PE among other methods. In summary, SL (${\text{T}\text{D}-\text{A}\text{U}\text{C}}_{\text{m}\text{o}\text{d}\text{e}\text{l} \text{I}}$ = 94.34; ${\text{C}-\text{i}\text{n}\text{d}\text{e}\text{x}}_{\text{m}\text{o}\text{d}\text{e}\text{l} \text{I}}$ = 91.60; ${\text{T}\text{D}- \text{A}\text{U}\text{C}}_{\text{m}\text{o}\text{d}\text{e}\text{l} \text{I}\text{I}\text{I}}$ = 93.73; ${\text{C}-\text{i}\text{n}\text{d}\text{e}\text{x}}_{\text{m}\text{o}\text{d}\text{e}\text{l} \text{I}\text{I}\text{I}}$ = 92.81) has a better ability to model risk prediction, and the introduced clinical models did not have a potential impact on improving the learning of methods.

Figure 2 illustrates the TD-AUC (Area Under the Curve) plot for Models 1 and 3. As evident, up to day 1000, all four algorithms exhibit significant fluctuations, with the super learner and Cox-PH demonstrating less variability compared to the other two algorithms. Beyond this point, all four algorithms stabilize in their performance, with the SL in Model 1 showing nearly constant predictions. Notably, there is a noteworthy improvement in the predictive performance of the GBM algorithm over time. Specifically, at day 6000, in Model 1, it outperforms Cox-PH, and in Model 3, it surpasses the performance of the other three algorithms.

### Variable importance

We utilized Model 1 to identify the impact of variables on the deaths from cardiovascular diseases, where all variables were in their standard scale. Relative influence is commonly used in regression models and machine learning algorithms to assess the impact of individual variables on the outcome of interest. According to Table 5, in the Cox model, age, waist-to-hip ratio, smoking status, systolic blood pressure, diastolic blood pressure, 2-hour blood glucose, and body mass index collectively determine more than 90% of the overall impact on attributed deaths from cardiovascular diseases. In the GBM model, age, 2-hour blood glucose, systolic blood pressure, smoking, aspirin use, diastolic blood pressure, and type 2 diabetes together contribute to approximately 89% of the total impact on attributed deaths from cardiovascular diseases. Meanwhile, in the support vector machine model, only three variables—age, waist-to-hip ratio, and smoking status—account for more than 89% of the total impact on attributed deaths from cardiovascular diseases. Lastly, the super learner model, incorporating eight variables—age, systolic blood pressure, smoking, waist-to-hip ratio, diastolic blood pressure, 2-hour blood glucose, body mass index, and aspirin use—determines more than 84% of the overall impact on attributed deaths from cardiovascular diseases.

**Table 5:** The effect of variables on prediction of deaths from cardiovascular diseases based on variable importance measure

Variable	Cox-PH (%)	GBM (%)	SVM (%)	SL (%)
Age	34.80	38.98	49.06	31.59
Waist-to-hip ratio	21.95	2.09	24.79	8.83
Smoking status	15.78	7.35	15.44	8.91
Systolic blood pressure	5.59	11.74	1.59	10.07
Diastolic blood pressure	5.01	3.97	1.26	8.29
Fasting blood sugar	4.13	17.52	1.17	7.31
Body mass index	3.09	1.52	0.71	5.77
Type 2 diabetes mellitus	1.99	3.54	0.85	3.20
Aspirin	1.06	5.75	1.08	3.64
Physical Activity	0.98	2.17	0.54	1.97
Cholesterol	0.97	1.32	0.69	1.41
Triglyceride	0.91	0.96	0.61	1.01
High-density lipoprotein	0.87	1.15	0.66	1.07
Sex	0.83	0.84	0.33	0.94
Lipid Drug	0.75	0.46	< 0.001	0.19
Family History of Stroke	0.54	< 0.001	0.74	2.93
Marital Status	0.38	< 0.001	0.23	1.29
Anti-Hypertension Drug	0.22	0.63	0.01	0.15
Education	0.13	< 0.001	0.21	0.86
Beta-Blockers	0.09	< 0.001	0.01	0.34
Corticosteroid	0.08	< 0.001	0.01	0.23

**Fig. 3:**
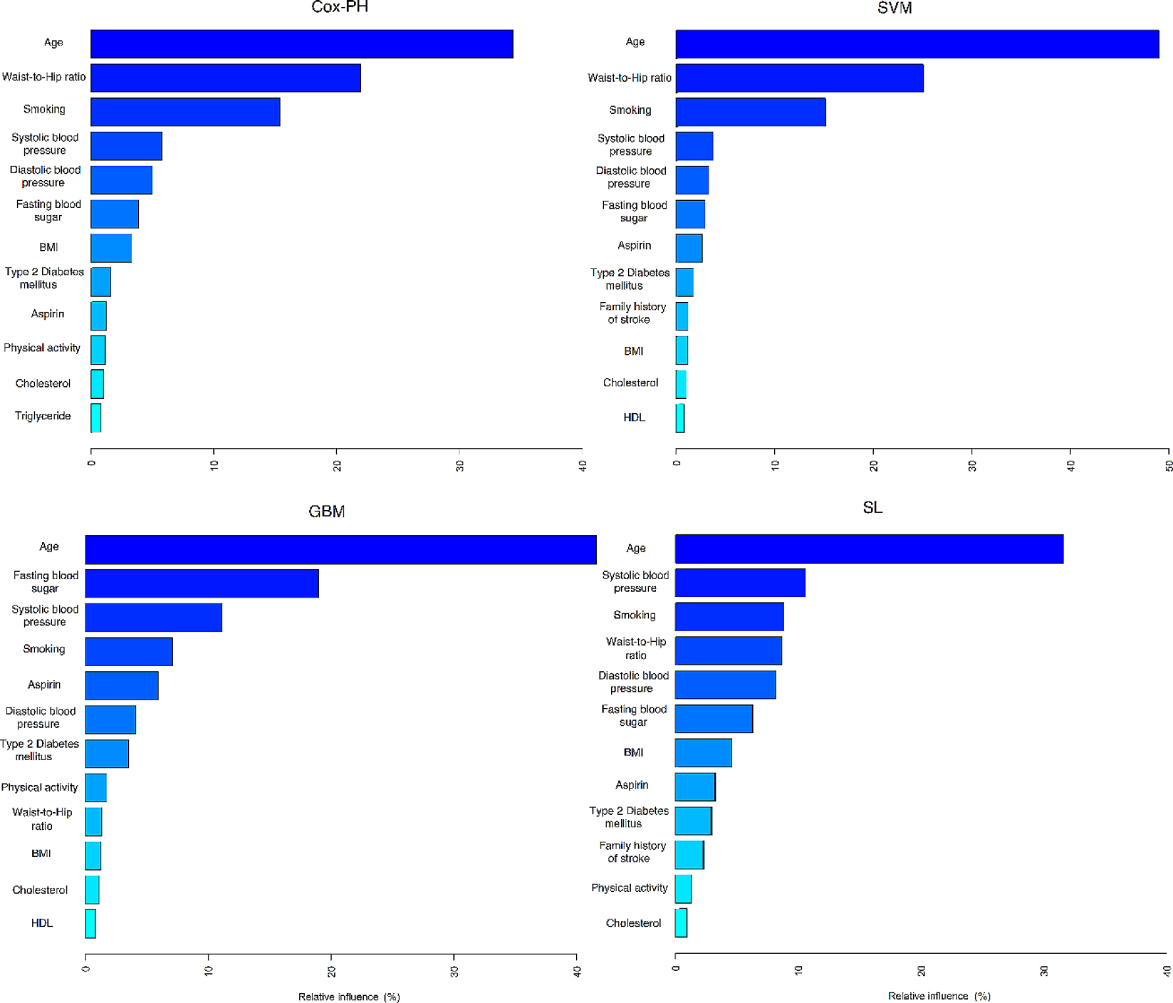
The top twelve variables with the highest Relative Influence on the performance of the Cox-PH, GBM, SVM, and SL algorithms. A higher value indicates a greater influence on prediction

Figure 3 illustrates the Relative Influence of the top twelve variables in four algorithms: Cox-PH, GBM, SVM, and SL. In each of these algorithms, age shows the highest impact on predicting CVD mortality.

## Discussion

In this study, we applied three distinct machine learning algorithms (GBM, SVM, and SL) alongside the traditional survival regression (Cox-PH) to predict CVD-related mortality in an urban population of 9258 participants from Tehran, Iran. To evaluate the models’ predictive performance, we compared risk predictions using metrics such as TD-AUC, c-index, BS, and PE. From a clinical perspective, our analysis identified age as the foremost risk factor for CVD-related mortality across all clinical models. Additionally, our study highlighted several crucial factors—FBS, SBP, waist-to-hip ratio, smoking, aspirin, anti-hypertension drug, DBP, T2DM, Hip, BMI, TG, HDL, and sex—as significant contributors to CVD-related mortality. We compared the models’ risk prediction performance using TD-AUC, c-index, BS, and PE.

Preliminary findings demonstrated TD-AUC and C-index exceeding 85% and 75%, respectively, for all models. The SL method emerged as the optimal model for identifying CVD-related mortality (TD-AUC = 94.34%; C-index = 91.60%). These results showed that, in TLGS, ML methods can match, and in the case of SL, even surpass the predictive power of the Cox-PH model. These finding align with various studies, supporting the effectiveness of ML approaches in predicting cardiovascular outcomes. For example, Hadanny et al. [58] compared survival prediction performance of Random Survival Forests and deep learning against Cox-PH in a set of acute coronary syndrome patients. They obtained a c-index of 0.95, 0.80, 0.80, and 0.83 for the random survival forest, deep learning, multivariate Cox-PH, and univariate Cox-PH, respectively. Heo et al., in a population of 2058 patients with coronary artery disease, showed that GBM can help identify hidden coronary artery disease in patients in long-term outcomes (TD-AUC = 76.3) [59]. Alaa et al., to evaluate the effectiveness of the most routine ML approaches in predicting the risk of CVD, showed that despite the same performance of ML-based methods, some methods such as GBM can better diagnose the disease. In their study Cox-PH, GBM, SVM, and random forest models yielded TD-AUC values of 75.8, 76.9, 70.9, and 73.0, respectively [10]. In another study, Torres et al. by comparing the performance of ML methods (Random Survival Forests, SVM, Extreme GBM) against Cox-PH in a set of 36,658 non-metastatic breast cancer patients, showed that Extreme GBM performed better (c-index > 70) and other methods predicting survival as good as Cox-PH (c-index > 60) [60].

To delved deeper into the performance of ML methods, we utilized four different clinical models, as detailed in variable definition and Outcome section. The results indicated an insignificant effect of clinical models on Cox-PH, GBM, and SL performance. However, this adjustment notably enhanced the SVM method’s performance, with clinical model III increasing TD-AUC by 5.72 units compared to clinical model I (${\text{T}\text{D}-\text{A}\text{U}\text{C}}_{\text{m}\text{o}\text{d}\text{e}\text{l} \text{I}}$ = 85.25; ${\text{C}-\text{i}\text{n}\text{d}\text{e}\text{x}}_{\text{m}\text{o}\text{d}\text{e}\text{l} \text{I}}$ = 75.99; ${\text{T}\text{D}-\text{A}\text{U}\text{C}}_{\text{m}\text{o}\text{d}\text{e}\text{l} \text{I}\text{I}\text{I}}$ = 90.13; ${\text{C}-\text{i}\text{n}\text{d}\text{e}\text{x}}_{\text{m}\text{o}\text{d}\text{e}\text{l} \text{I}\text{I}\text{I}}$ = 89.63). Feature selection’s importance is evident in several cross-sectional studies, emphasizing the need for a thoughtful approach to optimize model efficiency. For example, Rasheme et al. propose a model for feature selection in the early prediction of CVD by different ML methods [61]. In terms of survival studies, so far, no study has specifically covered this issue, but Alaa et al. used two clinical models (all variables and 7 core variables based on the Framingham cohort study) in examining the performance of routine ML methods against Cox-PH. According to the results of this study, it could be said that reducing the number of variables does not have a significant effect on the efficiency of the models [10].

The results of this study included two noteworthy points. First, the performance of all techniques was high in all models. One reason could be the small fraction of CVD-related mortality (228 of 9258 participants). Zhang et al. showed that in addition to traditional models, ML techniques have a high accuracy in the face of low event rates [62]. By creating samples with 0.12%, 10%, 20%, 30%, 40%, and 50% event rates, they showed that the SVM has the most sensitivity (${\text{a}\text{c}\text{c}\text{u}\text{r}\text{a}\text{c}\text{y}}_{0.12\%}$ = 99.88%; ${\text{a}\text{c}\text{c}\text{u}\text{r}\text{a}\text{c}\text{y}}_{50\%}$ = 73.23%) and the Bayesian network has the most insensitivity (${\text{a}\text{c}\text{c}\text{u}\text{r}\text{a}\text{c}\text{y}}_{0.12\%}$ = 64.43%; ${\text{a}\text{c}\text{c}\text{u}\text{r}\text{a}\text{c}\text{y}}_{50\%}$ = 70.53%) to the event rate. On the other hand, since the increase in data causes more knowledge of the techniques [63], another reason could be due to the small sample size (*n* = 9258) and the total number of features (dimension = 26). According to the above reasons, even though the event rate in Alaa et al. [10] study is 1.5%, but 473 features and 423,604 samples have had a significant impact on the results.

Second, the efficiency of all methods decreases in model IV compared to other clinical models. That could be due to overfitting in separating the dataset into training and testing data. In previous studies, it has been shown that the increase in potential risk factors causes overfitting and complexity of traditional models to obtain implausible results [16, 29]. On the other hand, in survival ML techniques, learning performs too long due to the presence of time. Too long learning is one of the most important factors in the formation of overlearning. In fact, overlearning was apparent in testing data [64]. So far, no survival studies have addressed the consolidation of overlearning, but Kassani et al. aimed to predict adolescent brain age based on multimodal sparse classification, introduced a redundant features pruning-based method that overcomes overlearning [65].

### Strengths

This article made a significant contribution to the field by applying advanced Machine Learning (ML) algorithm to predict Cardiovascular Disease (CVD) mortality using Tehran Lipid and Glucose Study (TLGS). The study stood out for its novel application of ML in addressing the pressing health concern of CVD in Iran, where the disease accounted for a substantial percentage of annual deaths and healthcare expenditure. The research employed meticulous methodologies, including comprehensive variable selection, innovative imputation strategies for handling missing data, and a thorough evaluation of four distinct models. Notably, the study not only emphasized the technical aspects of ML but also underscored the clinical relevance of its findings, identifying age as a consistent risk factor and highlighting crucial factors such as Fasting Blood Sugar (FBS) and Systolic Blood Pressure (SBP). The article’s strength lay in its rigorous evaluation of predictive models and its unique contribution to the understanding of CVD mortality in the Iranian population, making it a valuable addition to the literature on cardiovascular health prediction.

### Limitation

As previously mentioned, our study faced imbalanced data, with a minimal fraction of participants experiencing CVD-related mortality (approximately 2.5%). This imbalance led to TD-AUC values of the methods exceeding 90%. Hence, it’s crucial to assess the application of ML methods across diverse populations with varying event rates to ensure robustness and generalizability.

Furthermore, past studies have revealed correlations between functional factors like job stress and CVD occurrences. Integrating such information could provide a more holistic understanding of CVD outcomes within the TLGS (Tehran Lipid and Glucose Study).

Additionally, the ‘black-box’ complexity inherent in ML methods, especially in identifying linear interactions and independent effects on response variables in survival data, can pose challenges in interpretation due to potential overlearning. Bailly et al. highlighted how ML performance relies on the representation of the dataset’s original distribution and interaction terms. Hence, there’s a pressing need to develop methods that enhance data visualization and streamline redundant feature.

## Conclusion

Based on our analysis findings, the machine learning (ML) algorithm showed promising and occasionally superior performance in detecting CVD-related mortality compared to the Cox proportional hazards (Cox-PH) model. This observation was evident in a population-based study conducted among a diverse and sizable urban population in Tehran, Iran. Therefore, giving greater attention to ML methods could offer an automated mechanism for identifying patients who could benefit from preventive disease treatments.

### Electronic supplementary material

Below is the link to the electronic supplementary material.

Supplementary Material 1

## Data Availability

The datasets are not publicly available because these data are only available for approved proposals at Research Institute for Endocrine Sciences (RIES) in Shahid Beheshti University of Medical Sciences but are available from Davood Khalili, head of Department of Biostatistics and Epidemiology at RIES (email: dkhalili@endocrine.ac.ir) on reasonable request.

## Abbreviations

CVD: cardiovascular disease
Cox-PH: cox proportional hazard
ML: machine learning
GBM: generalized boosting machine
SVM: support vector machine
SL: super learner
WHO: world health organization
C-Index: concordance index
BS: brier score
PE: prediction error
AUC: area under the curve
TLGS: Tehran lipid and glucose study
SBP: systolic blood pressure
DBP: diastolic blood pressure
BP: blood pressure
BMI: body mass index
FBS: fasting blood sugar
DM: diabetes mellitus
T2DM: type 2 diabetes mellitus
TC: total cholesterol
TG: total triglyceride
HDL: high-density lipoprotein
