# Supplementary material for: Optimizing cardiovascular disease mortality prediction: a super learner approach in the tehran lipid and glucose study
Source: BMC Med Inform Decis Mak. 2024 Apr 16;24:97. doi: 10.1186/s12911-024-02489-0 (PMC11020797; doi:10.1186/s12911-024-02489-0)
Supplement: Supplementary file 1 — Supplementary Material 1 [file 12911_2024_2489_MOESM1_ESM.docx]

**Application of Machine Learning Methods in Modeling of Cardiovascular Disease Mortality: Tehran Lipid and Glucose (TLGS) Study: Supplementary**

**concordance index**

The concordance index, denoted as C-index, serves as a rank-correlation measure that evaluates the predictive accuracy of a model in the context of survival analysis. Specifically, it assesses the model's proficiency in correctly ordering the predicted survival times of pairs of subjects. In the realm of survival analysis, subjects are often individuals or items monitored over time to observe the duration until a specific event, such as failure or death, occurs [1].

The C-index utilizes a generalized Tau-Kendall correlation method tailored for censored data, providing a comprehensive evaluation of predictive performance. The index ranges from 0 to 1, where a value of 1 signifies exceptional model performance in distinguishing patients with differing outcomes (complete agreement), while a value of 0 indicates the model's inability to differentiate patients (no agreement) [2].

Mathematically, the concordance index can be expressed as the proportion of concordant pairs to comparable pairs [3]:

$C= \frac{Number of concordant pairs}{Number of comparabl pairs}$ ​

**Brier Score**

The Brier Score is a quadratic scoring function employed to assess the accuracy of predictions, particularly applicable to survival outcomes. It quantifies prediction accuracy by incorporating a weight function based on the conditional probability of uncensored observations over time [4]. The Brier Score, denoted as BS, ranges from 0 to 1, with values exceeding 0.25 indicative of suboptimal model performance, while lower values signify enhanced predictive accuracy [5].

Mathematically, the Brier Score can be calculated as follows:

$$BS= \frac{1}{N}\sum_{i=1}^{N} {(O_{i}-P_{i})}^{2}$$

In this formula:

- $N$ represents the total number of observations.
- $O_{i}$ denotes the actual outcome for the i-th observation.
- $P_{i}$ represents the predicted probability of the i-th observation.

**Integrated Brier Score**

The Integrated Brier Score is an extension of the Brier Score, designed to assess the overall accuracy of survival predictions over a specified time horizon. It combines the assessment of predictive accuracy across multiple time points, offering a comprehensive evaluation of a model's performance in predicting survival outcomes.

Mathematically, the Integrated Brier Score is calculated by integrating the Brier Score across all relevant time points. The formula for the Integrated Brier Score (IBS) is as follows:

$$IBS= \int_{0}^{T} BS(t)dt$$

Where:

- $T$ represents the endpoint of the time horizon under consideration.
- $BS(t)$ is the Brier Score calculated at each time point t.

The Integrated Brier Score provides a more holistic perspective on a model's predictive performance, capturing its consistency in accuracy across different time intervals. Lower values of the IBS indicate better overall predictive accuracy, demonstrating the model's effectiveness in forecasting survival outcomes across the entire time range of interest.

**Prediction Error**

In the evaluation of predictive models for survival analysis, the Integrated Brier Score (IBS) emerges as a comprehensive metric, providing a holistic assessment of predictive accuracy across various time points. To delve deeper into the temporal dynamics of our model's performance, we employ Prediction Error Curves based on the Integrated Brier Score.

**Time-Dependent Area Under the Curve**

The Time-Dependent Area Under the Curve (TD-AUC) serves as a pivotal metric in assessing the temporal predictive accuracy of machine learning algorithms. It quantifies the model's ability to discriminate between positive and negative outcomes at different time points during its prediction horizon. The formula for TD-AUC involves generating Receiver Operating Characteristic (ROC) curves at distinct time intervals and computing the area under each curve. Mathematically, TD-AUC is calculated as:

$$TD-AUC\left( t \right)=\int_{0}^{t} AUC(s)ds$$

Where t represents the specific time point, and $AUC(s)$ is the traditional Area Under the Curve calculated at time s. A TD-AUC value of 1 at a given time point indicates optimal predictive accuracy, showcasing the model's precision in discriminating outcomes at that moment.

**Cox proportionality assumptions**

Based on the evaluation metrics derived from the analysis, which include a Schoenfeld residuals test yielding a value of 0.10 and a p-value of 0.23 for the global test of proportional hazards, as proposed by Grambsch and Therneau [6], coupled with a thorough visual examination of residual plot demonstrating random dispersion, there is substantial evidence supporting the satisfaction of the proportional hazards assumption in the Cox Proportional-Hazards model applied to the TLGS dataset.

Ref.

1. Uno, H., et al., *On the C-statistics for evaluating overall adequacy of risk prediction procedures with censored survival data.* Stat Med, 2011. **30**(10): p. 1105-17.

2. Moncada-Torres, A., et al., *Explainable machine learning can outperform Cox regression predictions and provide insights in breast cancer survival.* Scientific Reports, 2021. **11**(1): p. 6968.

3. Fan, Y. and G. Yin, *Concordance index: Surrogacy of progression-free survival for overall survival.* Contemp Clin Trials, 2021. **104**: p. 106353.

4. Gerds, T.A. and M. Schumacher, *Consistent estimation of the expected Brier score in general survival models with right-censored event times.* Biom J, 2006. **48**(6): p. 1029-40.

5. Steyerberg, E.W., et al., *Assessing the performance of prediction models: a framework for traditional and novel measures.* Epidemiology (Cambridge, Mass.), 2010. **21**(1): p. 128-138.

6. Grambsch, P.M. and T.M. Therneau, *Proportional hazards tests and diagnostics based on weighted residuals.* Biometrika, 1994. **81**(3): p. 515-526.
